# Tickled to Death: Analysing Public Perceptions of ‘Cute’ Videos of Threatened Species (Slow Lorises – *Nycticebus* spp.) on Web 2.0 Sites

**DOI:** 10.1371/journal.pone.0069215

**Published:** 2013-07-24

**Authors:** By K. Anne-Isola Nekaris, Nicola Campbell, Tim G. Coggins, E. Johanna Rode, Vincent Nijman

**Affiliations:** 1 Nocturnal Primate Research Group, Oxford Brookes University, Oxford, OXON, United Kingdom; 2 Department of Software Development, Sonicated LTD, Oxford, OXON, United Kingdom; University of Kent, United Kingdom

## Abstract

**Background:**

The internet is gaining importance in global wildlife trade and changing perceptions of threatened species. There is little data available to examine the impact that popular Web 2.0 sites play on public perceptions of threatened species. YouTube videos portraying wildlife allow us to quantify these perceptions.

**Methodology/Principal Findings:**

Focussing on a group of threatened and globally protected primates, slow lorises, we quantify public attitudes towards wildlife conservation by analysing 12,411 comments and associated data posted on a viral YouTube video ‘tickling slow loris’ over a 33-months period. In the initial months a quarter of commentators indicated wanting a loris as a pet, but as facts about their conservation and ecology became more prevalent this dropped significantly. Endorsements, where people were directed to the site by celebrities, resulted mostly in numerous neutral responses with few links to conservation or awareness. Two conservation-related events, linked to Wikipedia and the airing of a television documentary, led to an increase in awareness, and ultimately to the removal of the analysed video.

**Conclusions/Significance:**

Slow loris videos that have gone viral have introduced these primates to a large cross-section of society that would not normally come into contact with them. Analyses of webometric data posted on the internet allow us quickly to gauge societal sentiments. We showed a clear temporal change in some views expressed but without an apparent increase in knowledge about the conservation plight of the species, or the illegal nature of slow loris trade. Celebrity endorsement of videos showing protected wildlife increases visits to such sites, but does not educate about conservation issues. The strong desire of commentators to express their want for one as a pet demonstrates the need for Web 2.0 sites to provide a mechanism via which illegal animal material can be identified and policed.

## Introduction

The internet is becoming the medium of choice for wildlife traders [1], [2], increasing not only the risk of over-exploitation of wild populations and disease transmission [3], but also the introduction of species outside their native ranges, where they may hybridise with native populations [4], [5]. Such internet trade may particularly affect Asian wildlife as Asia is a region disproportionately rich in threatened and restricted-range species [6], and where over 1 billion internet users – nearly 45% of global access – reside [7]. Many threatened species or their body parts are explicitly advertised for sale on websites, including charismatic Asian flagship species such as elephants, tigers, and marine turtles [8]. Pet traders increasingly resort to the internet, e.g. in Thailand [9] and in China [8]. Improved packaging and infrastructure allow ornamental fish and corals to be ordered on the internet and shipped to one's door in a matter of days. Internet trade is also used to offer legally protected species for sale as pets [8], [9]. For some species, like Iranian Kaiser's spotted newt, rising demands in internet trade of live specimens has seen an increase in their harvesting from the wild, leading to near extinction of the species [10].

Just as it has been shown that Web 2.0 sites may be used to promote products that have advertising bans [11], they also may be used indirectly to advertise exotic animals as pets by promoting the keeping of such animals. Web 2.0 sites provide online platforms for people to interact and collaborate to share interests, activities, backgrounds, or real-life connections by allowing users to share their personal details, information, ideas and imagery [12]. Numerous studies have alluded to the impact that Web 2.0 sites can have on public opinion [13]–[19]. Such opinions extend to conservation issues [14] when public outcry ultimately led to the internet auction site, Ebay, curtailing trade in protected species and rare valuables. Over the last eight years, YouTube has become one of the most popular online video sharing sites, and as of November 2012 was the third most-accessed site on the internet. Along with being a media sharing site, YouTube shares numerous features with social networking sites, in that users must sign up with a profile, and can then upload videos and/or create channels, subscribe to peoples’ video feeds, and can create comments or comment on threads on videos. It has become an important forum for exchange of public information [11], [15]. YouTube is policed by the public, and despite allowing the public to designate certain videos as ‘animal abuse,’ no option is available to viewers to designate when material depicting animals in videos is illegal.

With such an ability to reach the public, Web 2.0 resources such as YouTube are amongst the most powerful media for increasing awareness of conservation. For example, Web 2.0 platforms have been used to rally community support for local conservation initiatives [16]; for managing water sheds and implementing initiatives [17]; to encourage individuals to reduce their energy consumption [18]; and to fund raise for wildlife conservation [19]. At the same time, images presented by media can have damaging effects if viewers do not know the context. Ross et al [20] for example revealed that viewers presented with images of chimpanzees (*Pan troglodytes*) in anthropogenic contexts, such as standing next to a human, were more likely not only to want one as a pet, but also to assume that wild populations were not threatened. They noted that their results were “reminiscent of well-established marketing effects in the consumer behaviour community. The manner in which objects and products are displayed can have powerful impacts in the ways they are perceived, and consequently the value of objects can be greatly enhanced or degraded by the way in which it is presented [20, p. 5].” This potential for misperception suggests that conservation efforts may be hampered by images that are otherwise seemingly harmless.

The slow lorises (Lorisidae, *Nycticebus*) are a group of species made particularly well-known via Web 2.0 technologies, including YouTube. Over the last five years, videos of slow lorises kept as pets have become increasingly popular [21]. Eight species of slow loris, small nocturnal primates, are recognised; all are threatened, with the Endangered Javan slow loris (*N. javanicus*) being considered amongst the top 25 most endangered primates in the world. Major threats to slow lorises include: habitat loss, use in Asian traditional medicines, and capture for the photo prop and pet trades. Illegally-traded animals routinely have their anterior teeth inhumanely clipped out by vendors, a practice that inhibits release of animals back to the wild and often results in their death [21], [22].

Since 2007, all slow lorises have been listed on Appendix I of the Convention on International Trade in Endangered Species of Wild Fauna and Flora (CITES). Prior to this transfer, legal import and export occurred with approximately 2,000 slow lorises reported as traded between 1977 and 2004 [23]. Between 1975 and 2007 (i.e. the period when slow lorises were on Appendix II) some 1500 slow lorises, including 1330 live individuals, were imported by CITES Parties. Between 1975 and 2011 some 425 slow lorises were confiscated by importing Parties, including 245 live individuals. Most of these confiscations (171 individuals) concerned slow lorises being exported from Hong Kong into China [24], [Nijman, unpub.]. Difficulty in identifying these cryptic nocturnal primates, ease in smuggling them, and known volumes in rescue centres outside their range countries, mean that these numbers are likely drastically under-reported [25], [26]. Other underestimates come from lack of non-reporting; for example no confiscations are reported by Japan, a CITES member, despite many slow lorises being confiscated there [27].

Uploaded first in 2009, a video of the pygmy slow loris *N. pygmaeus* went ‘viral,’ becoming an internet sensation. The video features an overweight female, named Sonya, kept as a pet in a Russian flat being ‘tickled’ on a bed in a brightly-lit room. Based on coat colouration, fur density and the amount of white in the face, Sonya most resembles pygmy slow lorises as found in Vietnam. The uploader, Dmitry Sergeyev, included the following information in the original upload. “*1. First of all, we are located in St. Petersburg, RUSSIA. 2. It is NOT ILLEGAL in Russia to own this animals. 3. Our Sonya was born in a loris nursery and we have bought her in a local pet-shop. Thats why she is so tame and friendly*!” The Russian Federation became a Party to CITES on 1 January 1992, and prior to that the Soviet Union had been a Party since 1976. All range countries where pygmy slow lorises occur (China, Vietnam, Laos, Cambodia) are Party to CITES, and within each of these countries all slow loris species are protected by national laws. Hence in none of the range countries is trade in pygmy slow lorises allowed, and all commercial trade between the range countries and Russia (or formerly the Soviet Union) has either been strictly regulated (1975-early 2007) or banned (late 2007 – present).

When CITES Parties export species included on one of the Appendices of CITES they are obliged to report this to the CITES Secretariat and likewise when Parties import these species (be it from other Parties or from countries/territories not signatory to CITES) they need to report this to the CITES Secretariat. These records are maintained by and freely available from the UNEP-WCMC [28]. This being the case, it can be seen that in 1985, two pygmy slow lorises were imported into the (then) Soviet Union from Vietnam for scientific purposes, followed in 1989 by two more (included in the database as *N. coucang*, a species that does not occur in Vietnam) for a zoo. In 1991, three more pygmy slow lorises were imported, two from Germany and one from Sweden, all to be kept in a zoo. In 1998 and 1999, six and 11 pygmy slow lorises were illegally imported into Russia from Vietnam, and reported as such by the authorities. As such, in the last 27 years, five or seven pygmy slow lorises have been imported legally into the former Soviet Union and Russia, the most recent ones over 20 years ago. These individuals were imported to build up or to strengthen zoological collections, and none has been imported for commercial (trade) purposes. Many more have been imported illegally.

Slow lorises are difficult to care for in captivity as they are extremely sensitive to environmental stresses and even with specialised knowledge of species' ecology, mortality rates are high [21]. As of 2012, 56 zoos included in the ISIS database include pygmy slow lorises (*N. pygmaeus*) in their collection, two of them in Russia (Moscow and St Petersburg). Combined these zoos hold 202 individuals (including 5 in Moscow and 2 in St Petersburg). For the last 12 months, 7 out of these 56 zoos report breeding with a total of 11 offspring produced.

While it is difficult to comment whether or not Sonya and indeed her parents have been bred in captivity or whether they were obtained from the wild, it is clear that few individuals have been imported into Russia following CITES procedures. Thus there is a high likelihood that Sonya is derived from illegally imported and probably wild-caught pygmy slow lorises, or that she herself was derived from the wild.

There is an urgent need to quantify the impacts the role the internet has on wildlife trade by recording user behaviour and related attitudes that may lead to such behaviours. As current Web 2.0 technologies are in general user-policed, any additional information on species' threats currently must come from the public. As such, the comments provided in user-based forums provide a unique insight into the knowledge levels and perceptions of the public. The ‘tickling’ video provides a unique opportunity to explore and quantify these sentiments. We here address three key questions.

1. Do Web 2.0 resources increase public awareness of conservation issues facing a little-known threatened species, and can we quantify this by examining comments of a viral slow loris video?
2. Do YouTube videos of illegal slow loris pets incite the public's desire to purchase one?
3. Can celebrities' endorsements of slow loris videos impact public viewing of the videos?

## Methods

We analysed a single ‘tickling slow loris’ video, uploaded by wired.com on the 3^rd^ of June 2009. Shot in February 2009, the video was first uploaded on vimeo.com in the first week of February by Dmitry Sergeyev. After the first nine weeks and 13,000 visits, the video went viral, with 52,000 hits by the third week of April 2009 and 149,000 in the week following. At the end of April Mr Sergeyev uploaded the video onto YouTube. After 309,000 hits on vimeo.com, on the 3^th^ of June the video was uploaded on YouTube by wired.com, and despite other copies existing (Table 1), wired.com's YouTube post became the main site where the video was viewed. Wired.com removed the video on 22^nd^ of January 2012, at which point the video had generated 9,338,000 views and 12,411 comments.

**Table 1:** Parallel sites.

Site	Uploader	Date Uploaded	Views			
			*Feb 2012*	*July 2012*	*Feb 2013*	*June 2013*
vimeo.com video	Dmitry Sergeyev	February 2009	628,000	–	–	–
YouTube	Hamlollo	26 April 2009	768,491	1,923,714	3,389,778	3,978,700
YouTube	DmitrySergeyev	26 Apr 2009	71,582	297,478	347,069	376,283
YouTube	nw1024	19 Apr 2009	2	157,092	265,314	495,927
YouTube	wired.com	3 June 2009	9,338,000	–	–	–
YouTube	lillyblanchet	8 Jul 2010	7,893	119,968	161,361	191,756
YouTube	NickBShow	13 Jul 2010	10,000	14,478	17,237	17,518
YouTube	MrAlbell	20 Sep 2010	4,000	5,364	5,767	5,854
YouTube	SavasCelik	3 March 2013	–	–	–	347
Facebook	Alexandra Hay	7 April 2013	–	–	–	96,048
Facebook	Blake Sugar	8 April 2013	–	–	–	2,658

The video can be viewed on a large number of parallel sites; most of these are identical copies of the original, with some of them being several seconds shorter. Others show the video but with different types of background music. The dates below give an indication of virility of the video. When wired.com removed the video, Hamlollo became the main site to view it, and its viewing numbers soared; – indicates the video can no longer be viewed or was not yet uploaded.

We downloaded all 12,411 comments. The API (application programming interface) supplied by Google to download comments limits the comment download to the last 1000 records. Using the mobile website with AJAX (Asynchronous JavaScript and Extensible Markup Language) calls to retrieve 10 comments at a time in JSON format (Javascript Object Notation), we could retrieve all comments on the video. We incremented the number at the end of each URL (e.g. p = 200) in an automated process to retrieve all comments, parsed the JSON and output all data to a database for further analysis.

The interactive nature of YouTube videos allows for the unique analysis of content popularity and audience response [29]. Thus each comment was read and placed into one of the categories detailed in Table 2. Comments containing more than one category (e.g. “it's so cute – where can I get one?”) were scored twice. Commentators can post more than one comment, either in a string commenting on other posts or a separate post on different days. Each commentator can be identified by his/her unique user name (userID) (the number of commentators with more than one userID is assumed to be insignificant). For each month we calculated what proportion of commentators added a post on a specific topic (e.g. ”*slow lorises are poisonous*” or ”*slow lorises*' *bite is venomous*”). In almost every month >200 comments were posted, and common topics are presented on a monthly basis. We report all comments as direct quotes as written and spelled by users. Users upload information, such as sex, age, favourite books and music, and hobbies to their personal user profile for publication on YouTube. For each userID we downloaded further information about each user with the GData API [30], and this information was added to the database. To identify users with an interest in animals, we searched the “Hobbies” category for any reference to animals (these included keywords such as animals, animal rights, aquariums, badgers, bees, biology, butterflies, cats, cavies, dogs, dolphins, ecology, elephants, falconry, fennec foxes, ferrets, fish, horses, koalas, nature, penguins, pets, rabbits, reptiles, turtles, veterinary medicine, zoology).

**Table 2:** Categories into which we placed comments, example comments for each type, and total number.

Category	Example	Number
1. comment on behaviour; what loris is saying	“Awww when she stops tickling he looks SO sad!”	1281 (10.3%)
2. where can I get one/I want one	“I think that the slow loris is so cute: ') I want one, I don't care if they are illegal, I want one: D; this is THE CUTEST thing I have ever watched in my whole entire life. Every time I'm in the foulest of moods I will watch this and die over and over again. I WANT.”	1394 (11.2%)
3. cute, adorable, funny	“As far as I can see, as long as the animal is loved and happy, it doesn't matter. Providing they're treated right. I mean, obviously having wild animals isn't really right, but let's be honest... that animal would've already been shot or something for meat by now if it was in the wild... I think it's better off being tickled! And this video is adorable...it made me laugh!!”	2813 (22.7%)
4. illegal, threatened	“This is an endangered animal stolen from the wild at a young age, had its teeth pulled out with pliers, and is being kept in stressful conditions (lorises are nocturnal, and - rather than “loving” being tickled, this animal submits to being tickled as a passive defence-mechanism to deal with stress.) Please take a moment to flag this video and ask YouTube to remove it, as it contravenes their community guidelines (animal cruelty).”	641 (5.2%)
5. what is it?	“Okay I have to ask...what is it?? Lol it looks like a lemur but someone on my Facebook is saying it's a meerkat which I doubt.”	547 (4.4%)
6. Answer to what it is	“It's a Loris: D”	331 (2.3%)
7. factual (ecological, BBC documentary, domestication, toxin, dangerous)	“To people who are unsure, yes, the Slow Lorises are poisonous animals. A venom gland in their elbow is activated by them licking it. The venom then travels into their mouths and mixes with their saliva for a toxic bite.”	653 (5.3%)
8. lemur, Madagascar, any other film	“LOL he has a striking resemblance to King Julian from Madagascar.. ahaha too cute!”	310 (2.5%)
9. rant or very negative	“Maybe you should start reading another book, since the Bible is quite outdated.”	468 (3.8%)
10. Welfare (teeth pulling, not suitable pets, rehabilitation)	“The consequences of taking slow lorises from their natural habitat to sell them are often disastrous. Vendors pull their teeth with pliers and keep them in wire cages that tear their hands and feet. Between 30% to 90% of the animals die in transit.”	1192 (9.6%)
11. Other person directed them to the site	“That is adorable! I wonder how many views this gets thanks to Haley Williams?: P”	252 (2.1%)

Two key media events relevant to slow loris conservation occurred during the lifetime of the video. In March 2011, a video of a slow loris holding a cocktail umbrella went viral, reviving interest in loris pet videos. In response, A. Dunkel and K.A.I. Nekaris created a Wikipedia page entitled ‘Conservation of Slow Lorises.’ The authors of this page highlighted threats to slow lorises including habitat loss; pet, medicinal and bushmeat trades; described the process of teeth cutting; and also mention the loris as being venomous [31]. The second event was on the 25^th^ of January 2012, when an Icon Films production for the BBC *Natural World* programme entitled *Jungle Gremlins of Java* aired. This one-hour feature film specifically about the ecology and conservation of slow lorises not only reinforced the same messages as the Wikipedia page, but also contained gripping scenes of slow lorises being exploited in the pet trade. On the 9^th^ of February 2012, wired.com removed the ‘Tickling slow loris’ video, after nearly three years.

As an index of its virility, we examined celebrity endorsement of the video. These prominent public personalities typically had >50,000 and up to >5,000,000 Twitter followers and/or >500,000 and up to >250,000,000 views on their YouTube channels, qualifying them by our definition as celebrities (Table 3). When a celebrity was named in a comment or string of comments, we searched other Web 2.0 sources for the original comment made by the celebrity. We located seven of these original comments on Facebook, Twitter, YouTube, a celebrity’s personal blog or a combination thereof; one celebrity mentioned the video on a nationally-aired American television show. Where available, we recorded statistics on how often the comment was shared/retweeted or liked/ given thumbs up. We assume users who shared/retweeted or liked/ given thumbs up actually watched the video. While other celebrities may have made public endorsements about the video, by default, we restrict ourselves to those endorsements that led to at least one person making a comment.

**Table 3:** A summary of 252 celebrity endorsements, showing a continual flow of postings by celebrities as indicated by Date that the comment was made.

Celebrity	Occupation	Date Comment Made	NoTwitter YouTube	D2	Original Post	Reposts	Likes
Catrific	American YouTuber	Feb 2011	51,264; 8,282,575	3			
Miranda Cosgrove	American actress and singer-songwriter	July 2011	3,734,018; 5,892,924	22	“This video brought me an embarrassing amount of happiness...”	28	14
Justine Ezarik	American viral video comedian	Feb 2011	1,507,014; 285,164,901	1			
Stephen Fry	British entertainer	May 2010	5,724,090; n/a		“Those of you who champion the slow loris do have a point, of course”	107	54
Deidre Funk	American internet celebrity	Dec 2011	5,614; 1,067,893	4	“Lovers Gonna Love: I love hedgehog and slow loris videos on YouTube. You know you having a bad day or like maybe you're just bored. Just type into YouTube ‘Hedgehog Bath’ or ‘Tickling Slow Loris’ – so fricking cute. Most people think slow lorises are really gross. Which leads me to wonder why don’t I like birds but I do like slow lorises.”		
Ricky Gervais	British comedian and actor	April 2009	4,465,905; 14,693,313	6			
Ariana Grande	American actress, singer, and dancer	July 2011	5,800,923; 15,389,632	99	“So, @[] just showed me the cutest video in the world & I had to share it with you all asap. How cute? 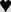 . ”	265	124
Tom Kaulitz	Tokyo Hotel – German Singer	July 2009	38,628; 72,367,871	22	“CERTAINLY NOT FOR YOUR HOME BUT SIMPLY IRRESISTIBLE I hate it when people rip animals out of their natural habitat just for their personal pleasure and for basically locking them up like a toy at home. But I NEED to show you this – whoever doesn’t go crazy must have a heart of stone. Slow Loris are the sweetest animals on earth but that’s also why they are doomed. Please also check this page and tell everyone you know about it. (links to conservation page).”		867
Shay Mitchell	Canadian actress and model	Nov 2010	1,244,333; n/a	1			
Josh Peck	American actor	July 2011	413,226; 435,669	1			
Betty White	American actress, Hot in Cleveland	December 2011	852,743; n/a	8	Mentioned on her television show *Hot in Cleveland*		
Hayley Williams	American singer	Feb 2010	80,474; 667,913	82	“Welcome to the changing of your life. (I dedicate the tweetage of this video to @hanngrenade)”		942
Drake Bell	American Musician and Actor	July 2011	1,623,625; 1,729,239	2			
Hungry Girl (Lisa Lillien)	Subscription service about healthy eating	July 2011	150,294; 18,053	1			

When the original post was available, we also present how often it was shared or retweeted (reposts), and how frequently it was ‘liked.’ NoTwitter YouTube =  how many Twitter followers and how many views the celebrity's YouTube channel had as of April 2013. Variable D2 = How many people were directed to YouTube specifically mentioning celebrities.

To examine the data we used various non-parametric statistical tests as implemented in SPSS 19.0. To measure differences in proportion of comments made about particular topics in each month, we used chi-square tests, testing outliers with the previous six months. To examine trends over time in the proportion of people making particular comments, we used Spearman's Rank Correlation Coefficient. We compared the distributions of specific topics, i.e. the monthly proportion of people that made the comment, with that of the monthly proportion of the total number of comments with a Kolmogorov-Smirnov two-sample test (two sample KS test). We set p at the 0.05 level, with all tests two-tailed; means are presented with standard deviations.

## Results

### Commentators

The 12,411 comments were made by 11,200 unique commentators between June 2009 and January 2012. On average, commentators post 1.26±0.09 comments with 91.9±0.01% of commentators making a single comment.

We were able to obtain user information from 72% of the 12,411 comments. These comments came from 172 countries. Commentators came mainly from North America (57%, with 4307 from US, 760 from Canada); EU (29%, n = 2539, 1337 from Great Britain, 407 from Germany); Australia and New Zealand (4%, 322). A smaller number, 21 (0.2%), were from Russia (the video's country of origin). Only 110 (1%) comments originated from nine of the 14 slow loris range countries: 35 from Singapore; 24 each from Malaysia and the Philippines, three from China and five from Vietnam. Although the age ranges were similar, more men made comments on the video (60% n = 4949, average age 27, range 13–97) than woman (40% n = 3595, average age 25, range 13–97). Of 2224 users who reported their hobbies, only 9.8% (n = 218) had an interest in animals (e.g. animals, nature, conservation, pets, horse riding), significantly fewer than commentators with other interests (χ^2^ = 1437.48, df = 1, p<0.0001).

### Content of Comments

We analysed 11 categories of comments, with a 12^th^ category of miscellaneous comments. Table 2 includes examples of the various types of comments in each of the 11 labelled categories, and the proportion of each type of comment made. The three most common types of comments referred to the animal being cute; the viewer commenting on what the animal was doing; or the viewer wanting one as a pet.

**Figure 1:**
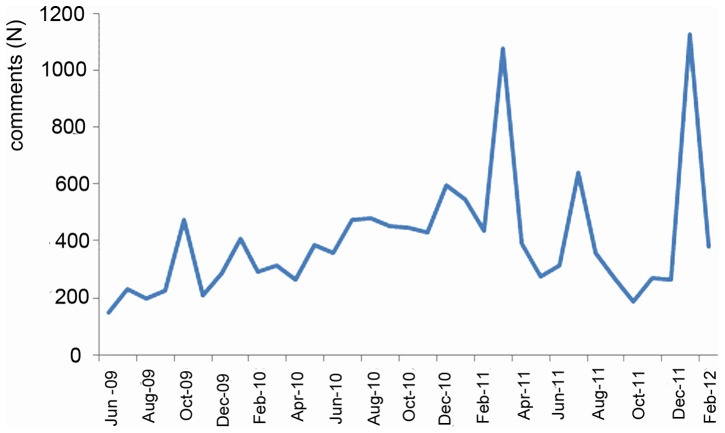
Number of comments made on a viral You Tube video . The relationship between number of comments and time; the two peaks coincide with the uploading of a second viral video of a slow loris holding a tiny cocktail umbrella and the production shortly thereafter of a slow loris conservation page on Wikipedia (March 2011) and the broadcasting of a BBC *Natural World* film on slow loris conservation, *The Jungle Gremlins of Java* (January 2012).

Two spikes, March 2011 and January 2012, occur in the viewing numbers of the video (Fig. 1). The total number of comments in both March 2011 (χ^2^ = 1175.0, df = 1, p<0.0001) and January (χ^2^ = 1359.1, df = 1, p<0.0001) was significantly higher than those made during all other months combined. The spike around March 2011 coincided with the appearance of the umbrella video and “Conservation of Slow Lorises Wikipedia” page. While this spike may not prove a causal relationship between appearance of the umbrella video and the Wikipedia page on the one hand, and an increase in comments on the other, 4.2% of the comments made during March 2011 specifically mentioned Wikipedia or the umbrella video (Wikipedia = 3.2%, umbrella = 0.8%, both = 0.2%). The second spike in January 2012 corresponded with the airing of *Jungle Gremlins of Java* and 13.3% of the comments made after its airing specifically mentioned this documentary, all occurring between the 26^th^ of January until the video's removal by wired.com on the 9^th^ of February.

**Figure 2:**
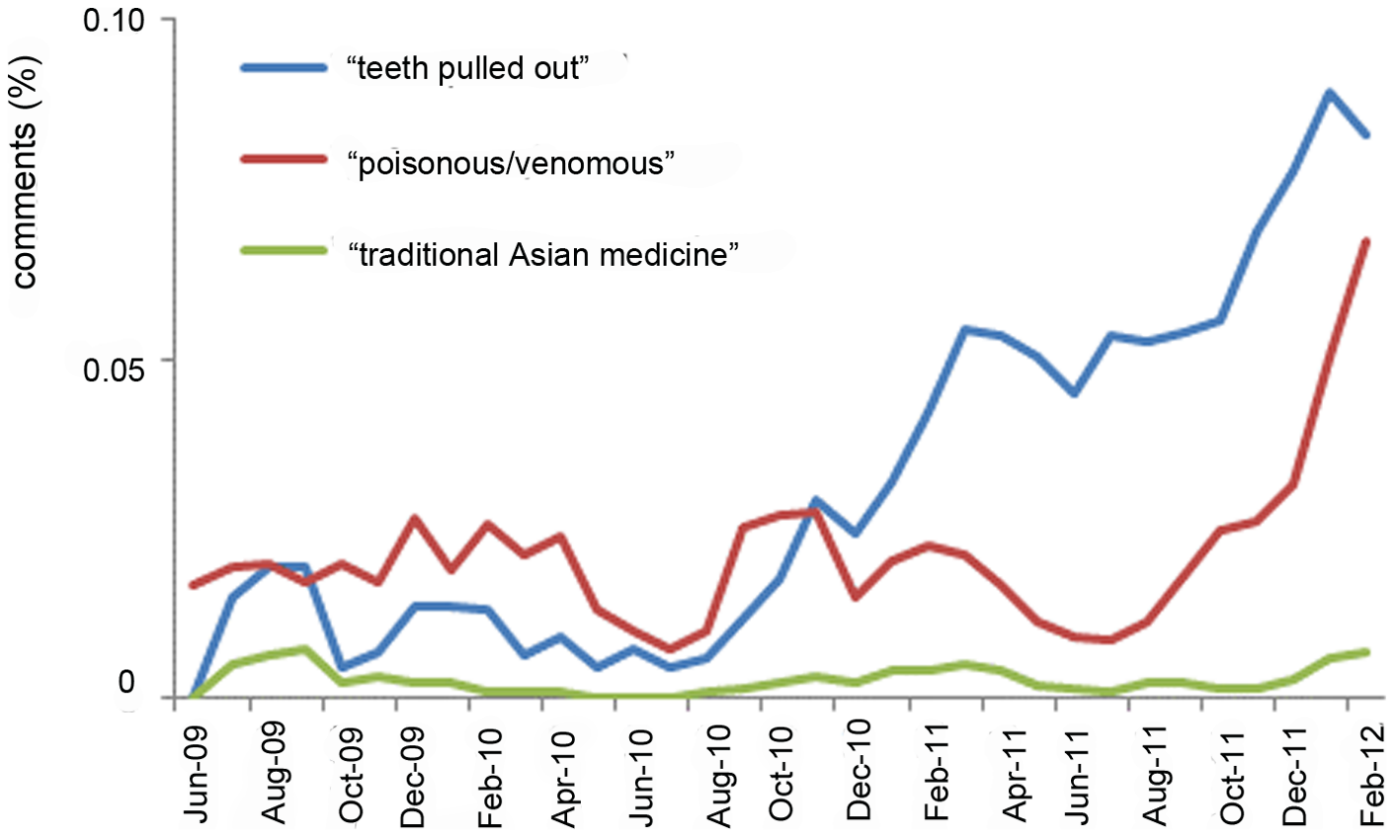
Increasing awareness over time. Indicated are the monthly proportion (3-monthly running mean) of commentators that mention specific facts about the slow loris biology or conservation status. Key: “teeth pulled out” refers to comments referring to the removal of slow loris' teeth in the illegal slow loris pet trade; “poisonous/venomous” refers to comments made about the venomous nature of the slow loris' bite and/or the species being poisonous; “traditional Asian medicine” refers to comments made referring to the use of slow lorises in (traditional) Asian medicine.

Many commentators felt that although the video contained illegal activity, it was increasing awareness. As one viewer put it, “*Great video. And contrary to what the sceptics say, this is a great form of awareness for the slow loris. Admit it, people. Before seeing this video, you didn't know what a slow loris was or the fact that it is an endangered species. Now you do*." In order to test this notion, we examined change in awareness over time. Although the video itself provided no educational material or web links, the comments themselves became a forum for providing such information. Over time, certain facts about slow loris conservation and ecology became more prevalent (Fig. 2). In particular, more viewers became aware of the disastrous consequences of slow lorises’ teeth being inhumanely removed in the pet trade (Spearman's Rank Correlation Coefficient, ρ = 0.730, n = 33, p<0.0001), with the proportion of commentators mentioning this in March 2011 being significantly higher than in all the months previous (χ^2^ = 27.0, df = 1, p<0.0001). However, the proportion of commentators who reported that a loris' teeth were ripped out in January 2012 did not significantly differ from the average number of people who stated this during previous months (χ^2^ = 0.36, df = 1, p = 0.55), despite a rising trend in this comment. At the same time, viewers made the link to the fact that slow lorises are the only venomous primates, and that their painful bite could make them unsuitable pets, but this trend was never significant (Spearman's Rank Correlation Coefficient, ρ = 0.040, n = 33, p = 0.823). Although the proportion of commentators who stated that a loris was venomous in March 2011 was not significantly higher than previous months (χ^2^ = 0.44, df = 1, p = 0.51), it tended towards significance after the January 2012 spike and the airing of *Jungle Gremlins of Java* (χ^2^ = 3.4, df = 1, p = 0.06). The other major threat to slow lorises, that of the traditional medicine trade, never became apparent in the comments despite its being one of the most critical threats to the pygmy slow loris (Spearman's Rank Correlation Coefficient, ρ = 0.168, n = 33, p = 0.35).

**Figure 3:**
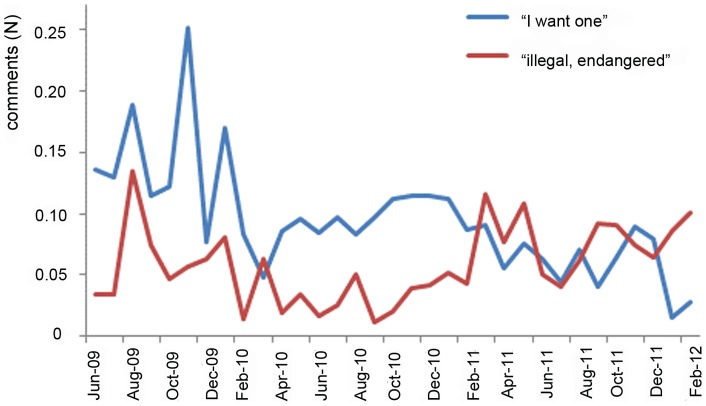
Comments on a viral YouTube video. Indicated is the monthly proportion of commentators that indicate that they wanted a slow loris as pet and those that indicted that it is illegal to keep slow lorises as a pet and/or that slow lorises are globally threatened. The proportion of commentators wanting a loris decreased significantly over time (Pearson's Product Moment Correlation, n = 33, R2 = 44.6%, p<0.0001). The proportion of commentators reporting the loris to be illegal/endangered did not increase significantly over time (Pearson's Product Moment Correlation, n = 33, R2 = 9.4%, p<0.08).

We examined viewers' desires to want a slow loris as a pet in relation to their awareness that it was illegal to have one (Fig. 3). Over time, more viewers began to comment on the fact that slow lorises are globally threatened (often using the term ‘endangered’) (Spearman's Rank Correlation Coefficient, ρ = 0.370, n = 33, p = 0.03). The increase became evident in March 2011, with more viewers stating that lorises were endangered than in the six months previous (χ^2^ = 45.5, df = 1, p<0.0001). This increase began to level off, and the proportion of commentators who stated that having a loris was illegal in January 2012 did not significantly differ from the average number of people who stated this during the previous six months (χ^2^ = 1.3, df = 1, p = 0.25). At the same time, the number of people wanting lorises as a pet dropped significantly from the initial reaction of around 25% of viewers wanting one, the majority of months saw about 10% of viewers wanting one (Spearman's Rank Correlation Coefficient, ρ = −0.710, n = 33, p<0.0001). This drop was *not* significant after the March 2011 spike, where the proportion of commentators who stated they ‘wanted’ a loris did not significantly differ from those who stated they wanted one during the previous six months (χ^2^ = 1.48, df = 1, p = 0.22). However, the proportion of commentators who stated they wanted a loris in January 2012 did significantly decrease from the average number of people who stated they wanted one during the previous six months (χ^2^ = 31.0, df = 1, p<0.0001).

The distribution over time of the proportion of commentators that referred to “teeth pulled out”, “poisonous/ venomous”, “traditional Asian medicine” (Fig. 2), “I want one”, or “illegal, endangered” (Fig. 3) did not differ from that of the distribution over time of the total number of comments (Fig. 1) (two-sample KS test, all KS>1.477, all p<0.025). The intensity of commenting thus did not have an effect on the topics that were discussed.

### Celebrity Endorsement

Comments were made by people directed to the site by 15 different celebrities on 252 occasions, where the celebrity was specifically named. Most celebrities posted the video on their blog, Facebook, or Twitter feed (or combination of those) with neutral comments or describing the ‘cute’ attributes of the loris, with Tom Kaulitz directing his viewers to conservation-related material (Table 3).

The impact of this varied. Most commentators coming to the site as a result of celebrity endorsements wrote neutral responses (75%), referring just to the celebrity, e.g. “*I*'*m so glad Ariana Grande sent me here!*” Many others thought lorises were cute (19%), or combined ‘cute’ with having been sent to the site by the celebrity. “*Thanks Ricky Gervais for sending me over to this clip, this is the cutest little being! He looks like he should be holding a sign. Perhaps the sign should say...check out rickygervais.com.*” Other commentators were inspired to have the loris as a pet (4%), such as the one who wrote “*Haley Williams sent me here. AND WHAT ANIMAL IS THIS? D: I WANT ONE, SO CUTE*.” Such comments carried on even beyond the initial posts. After Deidre Funk recorded a video describing how much she liked slow lorises, her fans corroborated her sentiments. One fan wrote on Deidre Funk's public Facebook site, “*So I'm convincing my mom to buy me a Slow Loris, if she legit lets me have one I'm naming it after you...: ')*” with Miss Funk replying, “*ahahha that would be amazing, i'd die xD*.” Only 2% mentioned conservation and that keeping slow lorises were illegal, all of which were in response to Kaulitz's anti-pet trade post.

## Discussion

Web 2.0 resources have introduced slow lorises to a public that would not normally come into contact with them. Although slow loris videos can serve to increase public understanding about some aspects of slow loris conservation, by the end of the airing of the analysed video, one in ten commentators still wrote that they wanted a loris as a pet. Our research provides data on public perceptions of media sharing websites related to keeping slow lorises illegally as pets, and here we discuss its implications.

Our study has three limitations, the first being that an important restraint of online studies is that they tend to be limited to a particular sample of the population. In our case, as indicated by our data, few commentators came from slow loris range countries. Secondly commentators may be attracted to animal videos in the first place and may already have a predilection to want an animal as a pet or to want to conserve an animal. We feel, however that this is not the case. Our data show that of more than 2000 viewers who listed their hobbies on YouTube, fewer than 10% had an interest in animals. The very nature of a video clip going ‘viral’ means that it quickly and exponentially reaches a very large cross-section of society that would not normally view it [13]. The nature of many of the comments was simply a naïve public learning what a slow loris was for the very first time. Secondly, we could have been biased in the coding of our content analysis, and considered some comments negative that were otherwise positive. We feel, however, for the purposes of what we illustrate here, that the results are robust, as most comments were quite short, and those who ‘wanted one’ were generally to the point, and those who wanted to point out a conservation message made it quite clearly (Table 2).

The power of YouTube to influence public opinions is now well-recognised. YouTube has become widely used as a tool by advertisers to sway consumer habits and in the public health industry to impact public lifestyle [29]. Web 2.0 sites, however, lie outside government regulation [32]. Increasingly their influence on human behaviour has been studied [11],[13],[18],[29],[32]. Many researchers, for example, have examined the impacts of Web 2.0 resources on public attitudes towards tobacco use [11], [29], [33]. Since many countries have now banned the advertising of potentially harmful products in the interest of public health, the internet has become the new mechanism of choice for advertising products such as tobacco [13]. Jenssen et al. [34] advocate the responsible use of anti-tobacco messages on search engines and on social networking sites where such ads are shown to counteract negative effects of the ads. If illegal loris videos cannot be removed from Web 2.0 sites, we whole-heartedly advocate a similar approach be adopted to inform viewers of the many reasons that dictate why slow loris trade is unsustainable, as outlined below.

Advertisers have long-recognised the power of celebrity endorsements to promote their products. Not only does having a celebrity associated with a product increase recognition and a positive attitude towards the product [34] but market researchers have also shown that celebrities can be used to increase sales of certain psychological or social high risk products such as alcohol [35]. Celebrity endorsements are meant to have a powerful psychological effect, as those viewing the endorsement are believed to follow a typical pattern, whereby they hope to identify with the celebrity, wanting to adopt their image. A second process is internalisation, whereby followers of the celebrity want to imitate the behaviour of that celebrity [36] [37]. ‘Tickling slow loris’ was seen at least 2400 additional times in our study due to celebrity endorsements via reposts (Table 3). In most of our examples, a celebrity simply microblogged a cute video without apparent knowledge of the legal or conservation implications of their post. Others encouraged their followers to get a loris as a pet. Other celebrities not included here actively maintain slow loris videos as permanent categories on their sites with comments on how cute they are. Permanent warnings embedded in videos of threatened species would allow the public to decide for themselves to agree with videos endorsed by celebrities. Furthermore, using the power of celebrities to communicate anti-pet trade messages via Web 2.0 technologies could be a powerful conservation tool.

**Figure 4:**
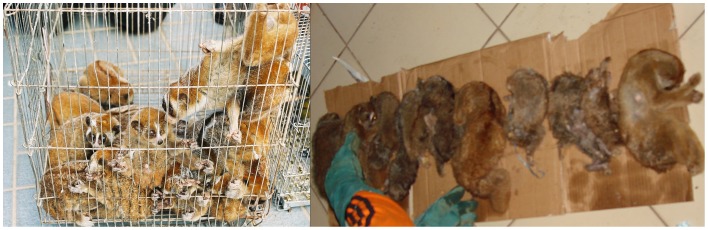
Slow loris trade has many impacts. Figure 4a shows a confiscation by Thai authorities of non-native pygmy slow loris (*N. pygmaeus*) en route for the illegal pet trade, which could potentially pose invasive species issues (Photo by Thai CITES Authority). Figure 4b shows a confiscation by Indonesian authorities of Sumatran slow lorises (*N. coucang*) en route to Java, all of which died, demonstrating that numbers at the end point are only an example of deaths that occur in trade (Photo by Dwi – WCS Sumatra).

Like other taxa impacted by wildlife trade that slowly reproduce [38], lorises produce only one offspring every one to two years in the wild after a 6–7 month gestation period, and survivorship is not guaranteed [39]. Captive reproductive success of lorises in accredited breeding facilities is extremely low, making it unlikely that lorises in pet shops come from commercial breeding facilities [40]. As has been shown time and time again in the illegal wildlife trade [41], it is far more likely that ”loris nurseries” and ”pet shops” serve as fronts for wild lorises smuggled from various habitat countries. Both *in-situ* and *ex-situ* death rate experienced by lorises in this trade, as evidenced by success in rescue centres and zoos, means that this slow-reproducing species cannot withstand this level off-take. For example, from a shipment of 102 pygmy lorises confiscated at a Taiwanese airport in 1993, more than 80% died between confiscation and arrival at their final destination at Saigon Zoo [42]. All pygmy lorises confiscated at Prague airport and entering quarantine of Prague zoo between 1990 and 2000 died [43]. Of 51 pygmy confiscated pygmy slow loris at Cuc Phong Rescue Centre in Vietnam, 15 (29%) died, eleven of which were juveniles of less than a year [44]. At International Animal Rescue in Indonesia, of 180 confiscated lorises from Java Sumatra and Borneo from 2008–2011, 64% had their teeth removed, eliminating any hope for reintroduction. Of those 180, 61 (34%) died despite intensive veterinary care [45]. Both the latter cases show not only the extreme susceptibility of these animals to stress in captivity, but also do not take into consideration the deaths of animals that died before confiscation took place (Fig. 4).

A simple decline in population of wild slow lorises is only one problem created by an increased demand for slow loris pets. Trade routes of lorises out of Asia into popular destinations of Eastern Europe, the Middle East and Japan have long been recognised [46]. For example, in 2012, pygmy slow lorises destined for use as pets in the Middle East and for use in the photo prop trade were confiscated in both Thailand and in India via Thailand [47]. Without intervention, practitioners may unwittingly release this species, not native to Thailand, into habitats containing the native *N. coucang* and *N. bengalensis* [46] [24]. The troubles of introducing non-native species from the fresh water aquarium trade have already been well-reviewed [48]. In some cases, smuggled animals carry highly pathogenic diseases [49].

Since the 2010 CITES I listing of slow lorises [23], trade in these species has not decreased. In fact, an increased number of international confiscations have been reported, more animals are being seen in domestic markets than ever before, more animals are coming into rescue centres, a photo prop trade in Thailand has boomed, and an increase in slow loris YouTube videos has occurred [Nekaris & Nijman, unpublished data], [TRAFFIC, unpublished data]. Alacs and George [4] caution that collectors may value species by their rarity and that both IUCN Red List and CITES classifications may be used by collectors to deem species as desirable, actually driving them to extinction. The viral nature of many slow loris videos, with animals portrayed as pets in a human setting, can serve to reinforce continually people's likelihood to want to acquire one [c.f. 20], [50]. In our study, despite the proportion of people wanting one as a pet statistically dropping, the number of commentators that wanted one remained high. Indeed, overall, “I want one” was the second-most frequently made comment (Table 1). This trend was further evidenced by the appearance of yet another new 2012 slow loris video, ‘slow loris eating sticky rice,’ where the uploader provided his viewers within the comments with information on pet shops and online sites in Japan from where they could buy a slow loris. Although few data are accessible in relation to *Nycticebus* trade on the Internet, investigations into slow loris availability show that Internet retailers based in Japan obtain animals from Indonesia and China [27]. Media sharing websites show a high volume of files pertaining to the slow loris as a pet, most of which also originate from Japan [Nekaris, unpublished data].

In our study we show the need for better regulations on media sharing websites such as YouTube. Currently most of these sites are only governed by the public and the owners of the sites can reap the financial rewards with a hands-off approach to the potential disastrous results of videos that imperil threatened species such as slow lorises. Better educational information is needed on websites that show protected species in illegal situations such as wildlife trade. The increase of specialist resources to ensure regular monitoring of media sharing should also be considered. YouTube, as a market leader, should set an example of international best practice in relation to wildlife trade.
