# Correction: Tickled to Death: Analysing Public Perceptions of ‘Cute’ Videos of Threatened Species (Slow Lorises – *Nycticebus* spp.) on Web 2.0 Sites

**DOI:** 10.1371/annotation/7afd7924-ca2b-4b9c-ac1b-2cc656b3bf42

**Published:** 2013-08-08

**Authors:** K. Anne-Isola Nekaris, Nicola Campbell, Tim G. Coggins, E. Johanna Rode, Vincent Nijman

The first author's name was incorrectly given in the byline and citation.

The correct name is K. Anne-Isola Nekaris

The correct citation is: Nekaris KA-I, Campbell N, Coggins TG, Rode EJ, Nijman V (2013) Tickled to Death: Analysing Public Perceptions of ‘Cute’ Videos of Threatened Species (Slow Lorises – *Nycticebus spp*.) on Web 2.0 Sites. PLoS ONE 8(7): e69215. doi:10.1371/journal.pone.0069215. 

